# Stress induces more serious barrier dysfunction in follicle-associated epithelium than villus epithelium involving mast cells and protease-activated receptor-2

**DOI:** 10.1038/s41598-017-05064-y

**Published:** 2017-07-10

**Authors:** Lei Zhang, Jun Song, Tao Bai, Wei Qian, Xiao-Hua Hou

**Affiliations:** Division of Gastroenterology, Union Hospital, Tongji Medical College, Huazhong University of Science and Technology, Wuhan, 430022 China

## Abstract

Psychological stress has been associated with intestinal epithelial hyperpermeability, the basic process in various functional and organic bowel diseases. In the present study, we aimed to clarify the differences and underlining mechanisms in stress-induced barrier disruption in functionally and structurally distinct epitheliums, including the villus epithelium (VE) and follicle-associated epithelium (FAE), a specialized epithelium overlaid the domes of Peyer’s lymphoid follicles. Employing an Ussing Chamber system, the epithelial permeability was assessed in rats following water avoidance stress (WAS) *in vivo* and in mucosa tissues exposed to corticotropin-releasing factor (CRF) *ex vivo*. Decreased transepithelial resistance (TER) and increased paracellular and transcellular macromolecular permeability in colon, ileal VE and FAE had been observed in WAS rats and in CRF-exposed mucosa. Especially, the barrier dysfunction was more serious in the FAE. Moreover, WAS upregulated the expression of mast cell tryptase and protease-activated receptor-2 (PAR2), which positively correlated with epithelial conductance. Mast cell stabilizer cromolyn sodium obviously alleviated the barrier disruption induced by WAS *in vivo* and CRF *in vitro*. Serine protease inhibitor aprotinin and FUT-175, and selective PAR2 antagonist ENMD-1068 effectively inhibited the CRF-induced FAE hyperpermeability. Altogether, it concluded that the FAE was more susceptible to stress, and the mast cells and PAR2 signaling played crucial roles in this process.

## Introduction

The human intestinal epithelium makes up the largest barrier, with a surface area up to 400 m^2^, separating the body from the luminal environment, such as toxins and antigens from food and microbes^1, 2^. Normally, only small amount of antigens could pass the intestinal barrier and play an important role in maintaining the immune function^1^. However, increased epithelial permeability may enhance the passage of antigens, facilitate the invasion of bacteria and lead to mucosal injury and inflammation. This process is the common pathophysiological basis of numerous organic and functional intestinal disorders such as inflammatory bowel disease and irritable bowel syndrome^3^.

Psychological stress is often suspected to adversely affect the intestinal barrier function^2^. It’s fact that, in gastrointestinal disorders, acute and persistent stress may induce/aggravate the gut dysfunctions and symptoms, impact the clinical remission and increase the risk of disease relapse^4^. The theory of brain-gut-axis has been well accepted which implicates a close relationship between the functions of the gut and the brain^5^. The bad emotions no doubt lead to negative sensation and mood of the gut. The mechanisms are complex and multifactorial, but at least including the neural and humoral pathways especially the autonomic nervous system and the hypothalamic-pituitary-adrenal axis^6^. Corticotropin releasing factor (CRF) is one of the most important stress hormones, produced in both the central nerve system and peripheral tissues, and has been shown to participate in stress-related gastrointestinal dysfunctions by acting on the CRF receptor type 1 and type 2 (CRF1 and CRF2)^4^. CRF could disrupt the intestinal barrier in multiple *in vivo* and *in vitro* researches^2, 7^. Several studies have suggested mast cells (MCs) as crucial effector cells of CRF in the gut, which express the CRF1 and CRF2 receptors and release multifunctional mediators such as proteases, histamine and cytokines upon activation^7, 8^. However, the precise mechanisms of stress-induced mast cell-mediated intestinal hyper-permeability remain poorly elucidated, particularly in functionally and structurally distinct regions of the gastrointestinal tract.

In most cases, assessment of intestinal epithelial permeability was focused on the villus epithelium (VE), but ignored the importance of follicle-associated epithelium (FAE)^1, 9^. The FAE is a specialized epithelium with distinct functional and structural features that overlays the domes of lymphoid follicles (Peyer’s patches) in the small intestine^10, 11^. The FAE typically contains numerous microfold cells which facilitates sampling and transporting luminal macromolecules and microorganisms to the underlying mucosal immune system^10, 11^. The epithelial permeability of the FAE obviously differs from the VE^10^, however, the differences in responses to psychological stress were rarely reported. Furthermore, systemic evaluation of the differences in stress-induced epithelial barrier disrupt among different bowel segments was required, since the intestinal barrier function varies in a segment-specific way^12^.

In this study, we aimed to assess the mechanisms and differences in stress-induced barrier dysfunction in structurally and functionally distinct epitheliums of the gut, including the colonic epithelium, ileal VE and FAE.

## Results

### Body weight, stool pellet, and visceral sensation

**Figure 1:**
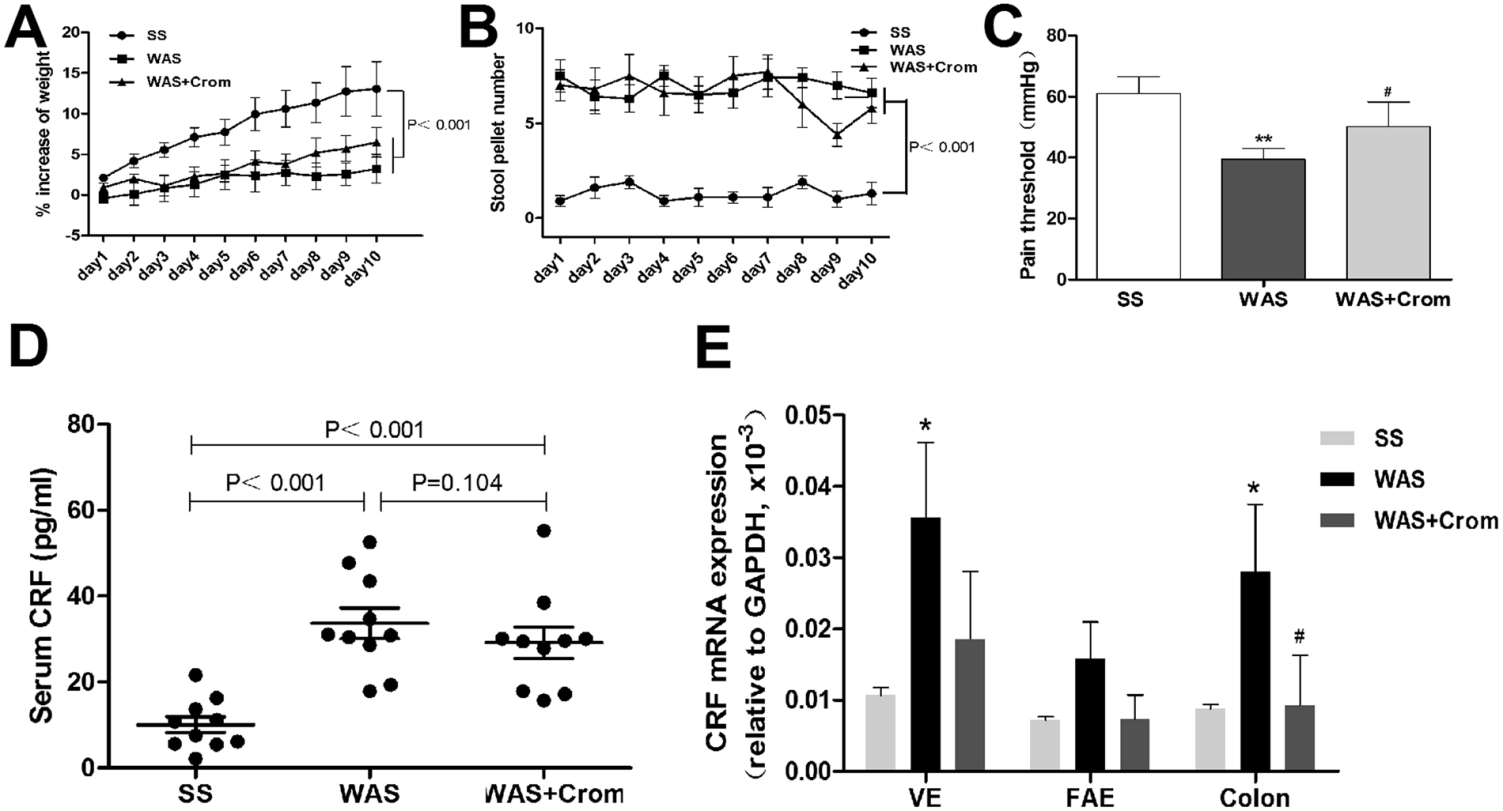
Effects of WAS on the body weight, stool pellet, visceral sensation, and CRF levels in serum and intestine. (**A**,**B)** WAS rats showed failure to thrive and stool pellet increasing compared with SS rats, mast cell stabilizer cromolyn didn’t influence on the weight gain and defecation during stress. **(C)** Decreased pain threshold in WAS rats and effectively improved in WAS + Crom rats. **(D)** WAS induced significant increase in serum CRF which didn’t inhibit by cromolyn. **(E)** mRNA expression of CRF in the colon, ileal VE and FAE enhanced during stress and partially reduced by cromolyn. N = 10 for each group. Data are displayed as the mean ± SD.

Chronic water avoidance stress (WAS) induced failure to thrive (Fig. 1A), increased stool pellet number during stress (Fig. 1B) and decreased pain threshold (Fig. 1C). Mast cell stabilizer cromolyn didn’t influence on the weight gain and defecation during stress, but effectively improved the pain threshold of WAS rats (Fig. 1A–C).

### Serum and intestinal CRF levels

The serum levels of CRF increased in WAS rats relative to the SS rats, while cromolyn didn’t inhibit the serum CRF increase induced by stress (Fig. 1D). Similarly, the mRNA expression of CRF were enhanced significantly in ileal VE and colon (P < 0.01), and there was an increasing trend in ileal FAE (P = 0.061). Cromolyn may decrease the CRF expression in the intestine especially for the colonic mucosa (Fig. 1E).

### Inflammatory scores

**Figure 2:**
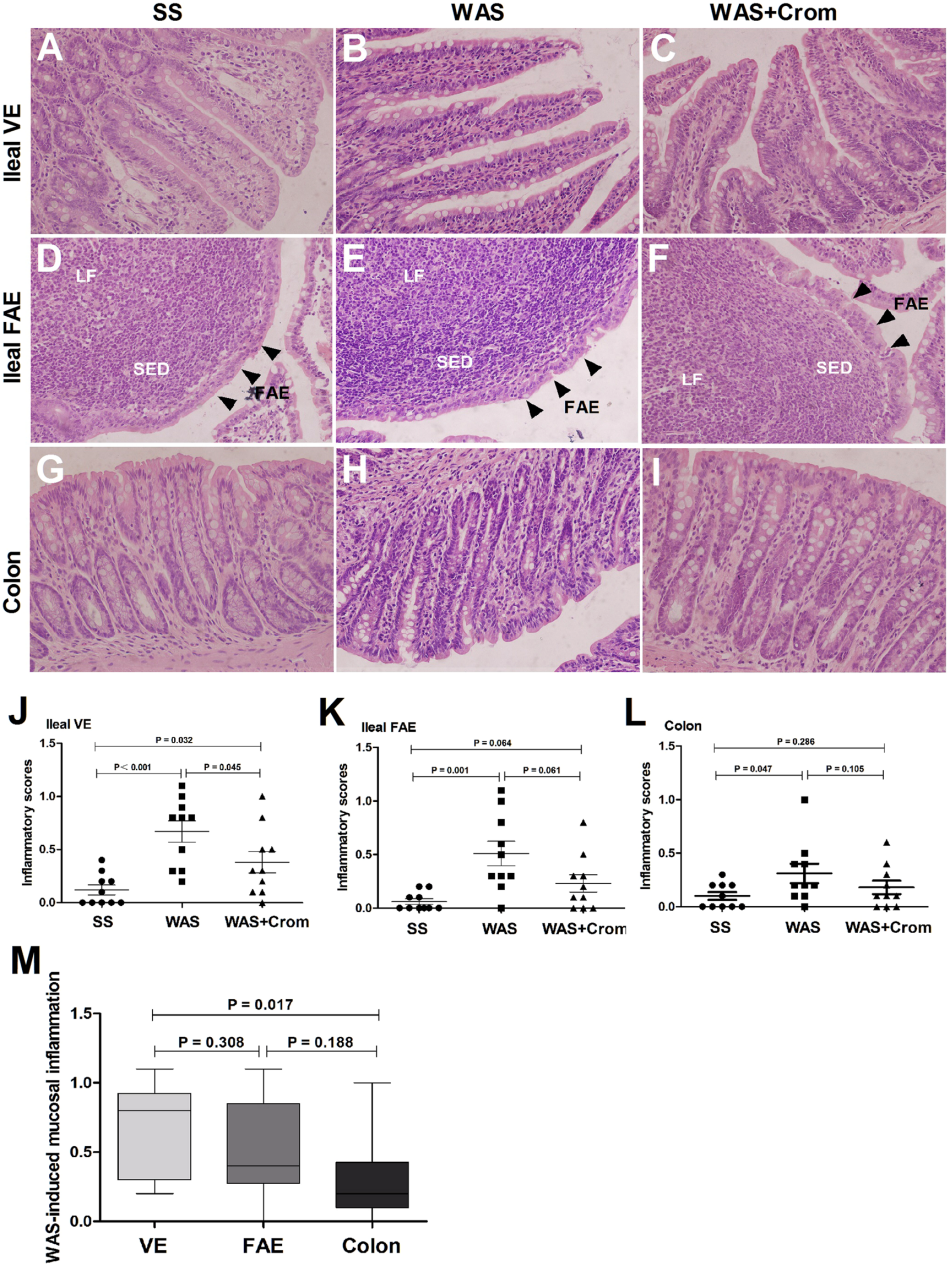
Effects of WAS on the inflammatory infiltration in the colon, ileal VE and FAE. **(A**–**I)** Typical hematoxylin-eosin staining of ileal VE, ileal FAE and colon form SS group, WAS group and WAS + Crom group, respectively. There were obviously inflammatory infiltration in WAS rats, and improved in WAS + Crom rats. **(J**–**L)** Histological scores in the colon, ileum VE and FAE were increased in WAS rats, and trended to be decreased by cromolyn treatment in WAS + Crom rats. **(M)** WAS induced more serious inflammation in ileal VE relative to colon. N = 10 for each group. Data are displayed as the mean ± SD.

There were obvious intestinal inflammation in the mucosa of colon, ileum VE and FAE in WAS rats with increased lymphocytes infiltration and histological scores. Cromolyn treatment trended to improve the mucosal inflammation induced by WAS, especially in the ileal VE and FAE (Fig. 2A–L). Relative to colon, the WAS induced more serious inflammation in ileal VE. However, there were no significant differences between FAE with VE or colon (Fig. 2M).

### Mast cell infiltration and tryptease/PAR2 expression

**Figure 3:**
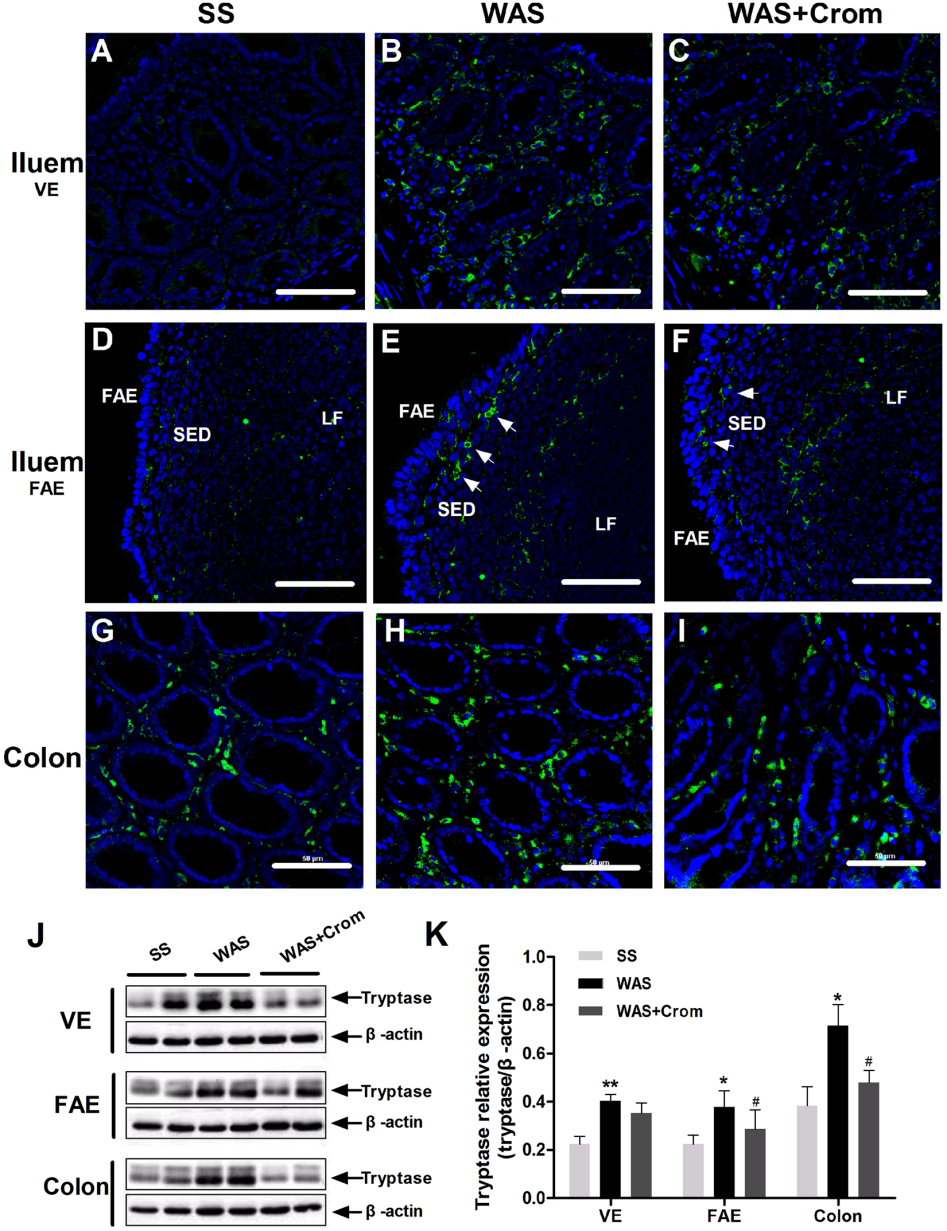
Effects of WAS on the expression of mast cell tryptase in the colon, ileal VE and FAE. **(A**–**I)** The tryptase^+^ cells increased in mucosal tissues of ileal VE, ileal FAE and colon in WAS rats, and decreased in cromolyn treated WAS rats. SED, subepithelial dome; LF, lymphoid follicles. Scale bars: 50 μm. **(J**,**K)** The expression of tryptase in colon, ileum VE and FAE were upregulated in WAS rats, and alleviated in WAS + Crom rats. N = 10 for each group. Data are displayed as the mean ± SD. *P < 0.05, **P < 0.01, compared with SS group; ^#^P < 0.05, compared with WAS group. followed by IBS-D FSN stimulation (**P < 0.01). The gels were run under the same experimental conditions. Cropped blots are displayed and full-length blots are included in the supplementary information.

**Figure 4:**
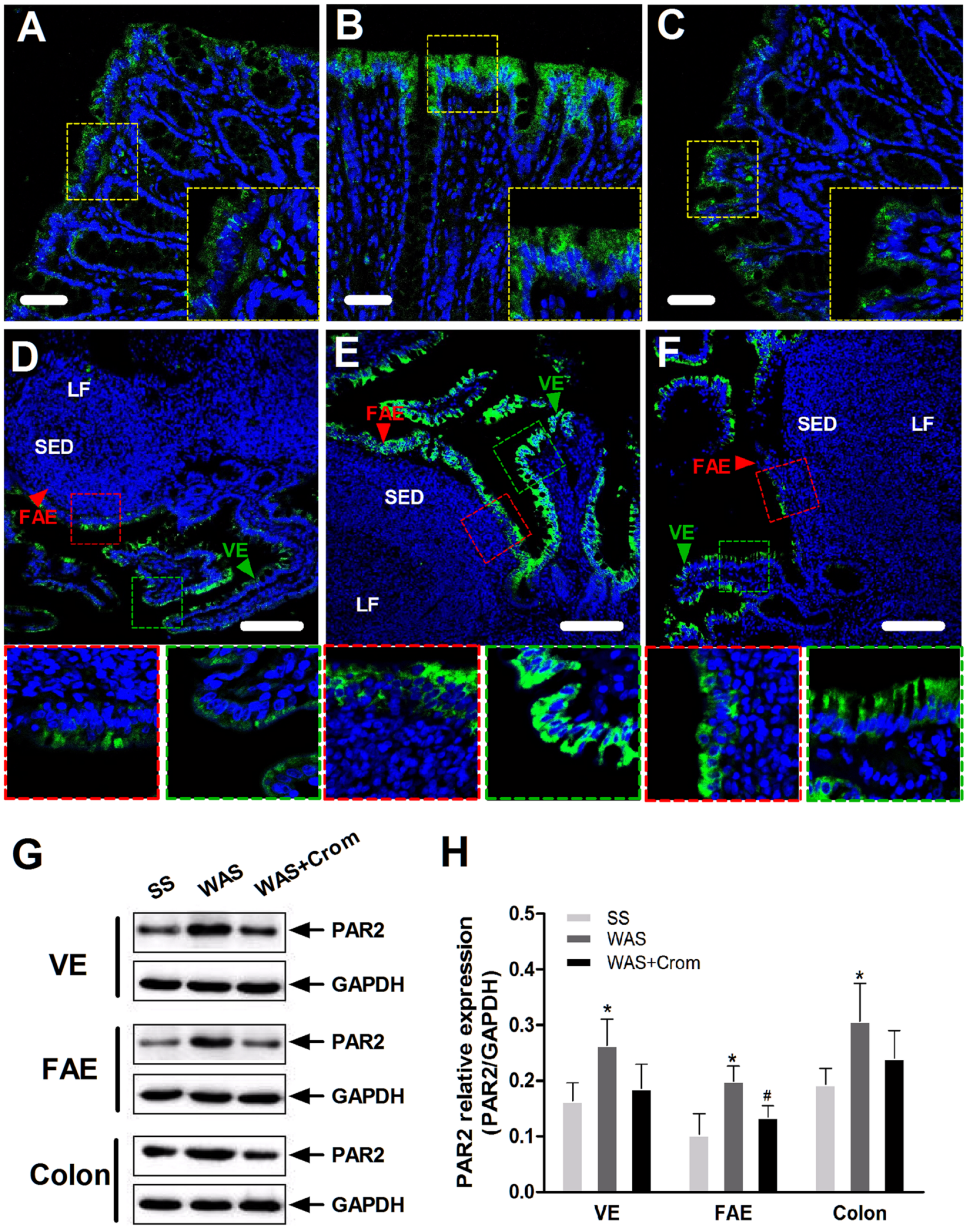
Effects of WAS on the expression of PAR2 receptor in the colon, ileal VE and FAE. (**A**–**F)** The PAR2 receptor was located on the epithelial layer of the colon, ileum FAE and adjacent VE, which upregulated in WAS rats, and may be downregulated by cromolyn. SED, subepithelial dome; LF, lymphoid follicles. Scale bars: 50 μm for (**A**–**C**) and 100 μm for (**D**–**F**). **(G**,**H)** Higher expression of PRA2 was observed in WAS rats in colonic epithelium as well as the ileum FAE and VE. Cromolyn treatment significantly reduced the levels of PAR2 in FAE. N = 10 for each group. Data are displayed as the mean ± SD. *P < 0.05, compared with SS group; ^#^P < 0.05, compared with WAS group. The gels were run under the same experimental conditions. Cropped blots are presented and full-length blots are shown in the supplementary information.

**Figure 5:**
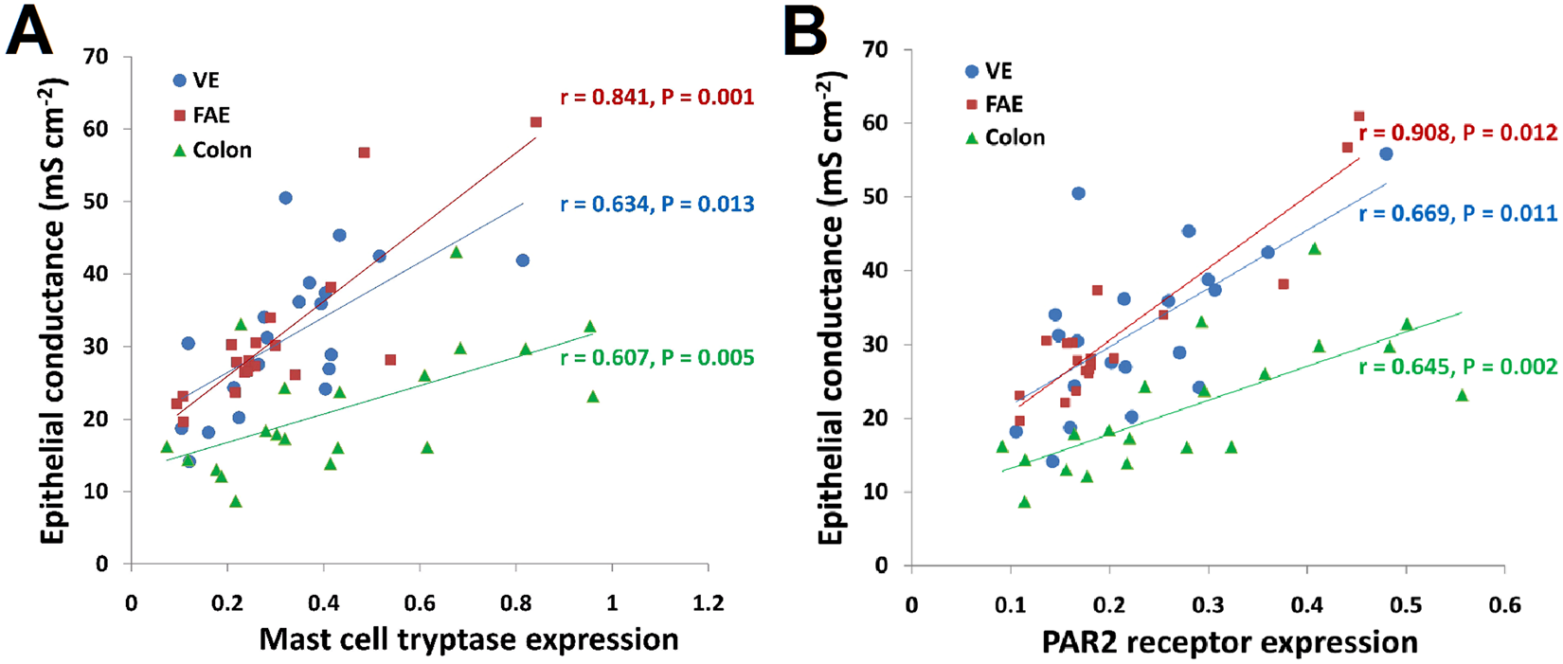
Positive correlations between mast cell tryptase/PAR2 and epithelial conductance in the colon, ileum VE and FAE. (**A**) Mast cell tryptase and (**B**) PAR2 receptor were positively correlated with epithelial conductance in the colon, ileum VE and FAE, which is more obviously in FAE. Data are displayed as the mean ± SD.

Mast cells, the tryptase^+^ cells, infiltration in mucosal tissues of ileal VE (Fig. 3A–C), ileal FAE (Fig. 3D–F) and colon (Fig. 3G–I) were increased in WAS rats, and decreased in cromolyn treated WAS rats. The expression of mast cell-derived tryptase in colon, ileum VE and FAE were obviously upregulated in WAS rats, and alleviated in those treated with cromolyn (Fig. 3J,K). The proteinase-activated receptor-2 (PAR2) was located on the epithelial layer of the colon, ileum VE and FAE. Higher expression of PRA2 was observed in WAS rats in colonic epithelium (Fig. 4A–C) as well as the ileum FAE and adjacent VE (Fig. 4D–F), which may be downregulated by cromolyn. It also was verified by the results of immunoblot, especially for the FAE (Fig. 4G,H). Furthermore, there were strongly positive relations between the levels of tryptase, as well as PAR2, and epithelial conductance in the colon, ileum VE and FAE, which is more obviously in FAE (Fig. 5).

### Stress-induced intestinal hyperpermeability was different in ileal VE, FAE and colonic epithelium

**Figure 6:**
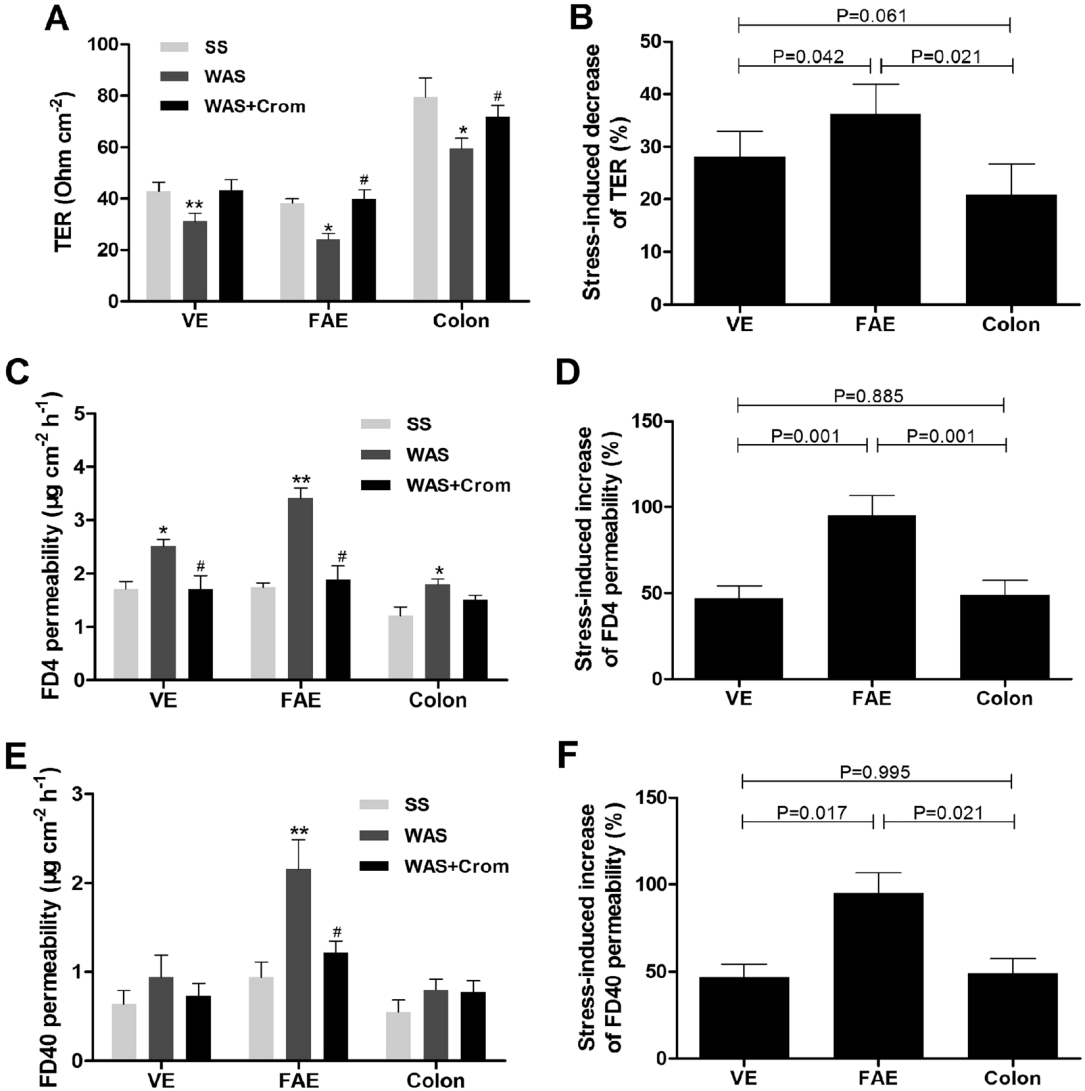
Stress-induced intestinal hyperpermeability was different in ileal VE, FAE and colonic epithelium. (**A**,**C**,**E**) WAS induced significant decline of TER and increase of paracellular FD4 permeability in ileal VE, FAE and colon, and FD40 permeability in ileal FAE, which could be partially blocked by cromolyn. **(B**,**D**,**F)** The ileal FAE was more sensitive to stress with higher decrease of TER and higher increase of FD4 and FD40 permeability relative to the ileal VE and colon. N = 10 for each group. Data are displayed as the mean ± SD. *P < 0.05, **P < 0.01, compared with SS group; ^#^P < 0.05, compared with WAS group.

Stress induced significant decline of transepithelial resistance (TER) and increase of paracellular permeability to fluorescein-isothiocvanate (FITC)-dextran 4 kDa (FD4) in ileal VE, FAE and colon, which could be partially relieved by cromolyn (Fig. 6A,C). However, the transcellular permeability to fluorescein-isothiocvanate (FITC)-dextran 40 kDa (FD40) was enhanced obviously just in ileal FAE and blocked by mast cell stabilization (Fig. 6E). Importantly, the ileal FAE was the most influenced region by stress with higher decrease of TER and higher increase of FD4 and FD40 permeability. There was no significant difference between ileal VE and colon (Fig. 6B,D,F).

### Differences in CRF-induced increase of permeability in VE, FAE and colonic epithelium

**Figure 7:**
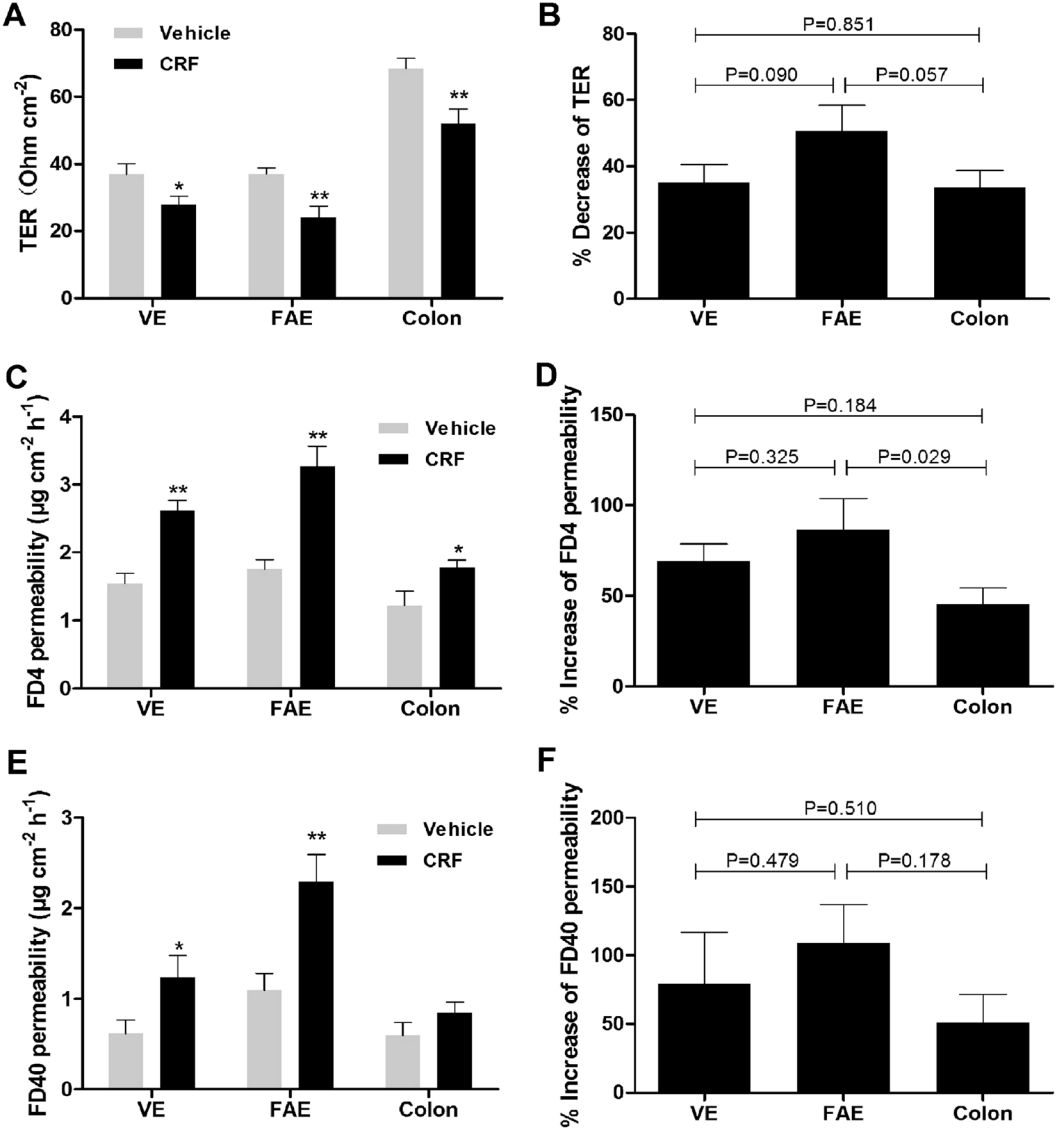
Differences in CRF-induced increase of permeability in ileal VE, FAE and colonic epithelium. (**A**,**C**,**E)** CRF exposure disrupted the barrier function of the ileal VE, FAE and colon, including TER decreasing and increased permeability to FD4 and FD40. **(B**,**D**,**F)** The TER decreasing and FD4 and FD40 permeability increasing were trend to be more serious in the ileal FAE relative to the ileal VE and colon, but part of them had no statistical significance. N = 12 for each group. Data are displayed as the mean ± SD. *P < 0.05, **P < 0.01, compared with vehicle.

CRF exposure mimicked the changes in epithelial permeability induced by stress, including TER decreasing and increased permeability to FD4 in ileal VE, FAE and colon, as well as hyperpermeability to FD40 in ileal FAE and VE (Fig. 7A,C,E). Similarly, the barrier disruption, such as TER decreasing and permeability increasing, was trend to be more serious in the ileal FAE relative to the ileal VE and colon, but most of them had no statistical significance (Fig. 7B,D,F).

### Mast cells and PAR2 mediate FAE barrier disruption by CRF exposure ***in vitro***

**Figure 8:**
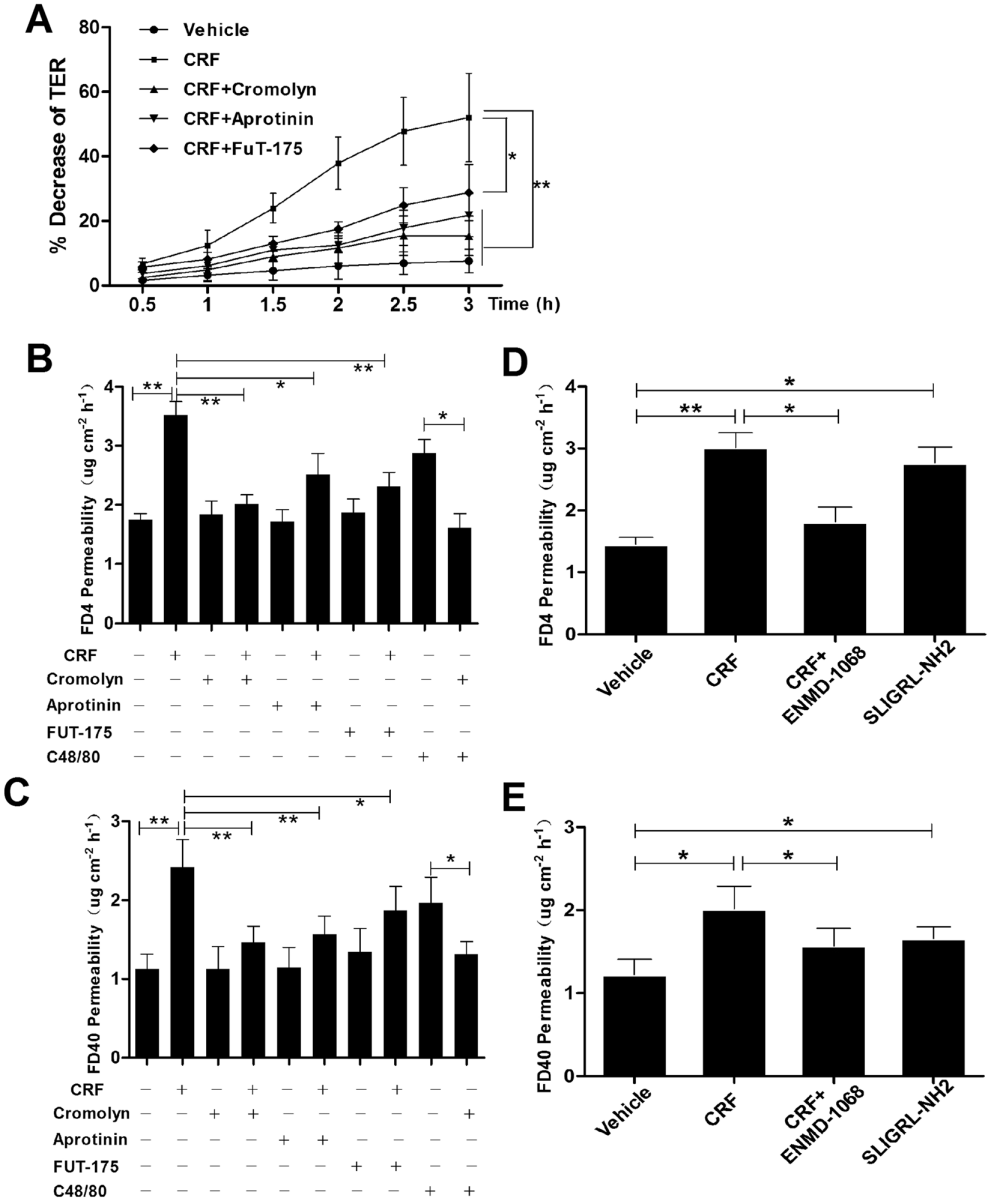
Mast cells stabilization and protease/PAR2 inhibition improved the FAE barrier disruption under CRF exposure *in vitro*. (**A**) CRF exposure led to notable decrease of TER in the FAE in a time-dependent manner, and markedly blocked by mast cell stabilizer cromolyn, protease inhibiter aprotinin and PAR2 antagonist FUT-175. **(B,C)** CRF significantly increased the FD4 and FD40 permeability, which may alleviate by cromolyn as well as aprotinin, FUT-175 and ENMD-1068. Compound 48/80, a mast cell activator, induced FAE heperpermeability to FD4 and FD40, and entirely blocked by mast cell stabilization. **(D,E)** Selective PAR-2 activation by SLIGRL-NH2 significantly increased both the FD4 and FD40 permeability of FAE. N = 12 for each group. Data are displayed as the mean ± SD. *P < 0.05, **P < 0.01.

The mechanisms underlined the FAE heperpermeability induced by stress were further studied. CRF exposure led to notable decrease of TER in the FAE in a time-dependent manner which could be markedly inhibited by cromolyn and partially blocked by protease inhibiter aprotinin and FUT-175 (Fig. 8A). The heperpermeability to FD4 and FD40 induced by CRF were also alleviated when pretreated with cromolyn as well as aprotinin, FUT-175 and ENMD-1068. Moreover, mast cell activation by compound 48/80 resulted in the FAE heperpermeability to FD4 and FD40, and entirely blocked by mast cell stabilization. Selective PAR-2 activation by SLIGRL-NH2 significantly increased both the FD4 and FD40 permeability of FAE (Fig. 8B–E).

## Discussion

In this study, for the first time, we mapped the different susceptibility of functionally distinct epitheliums to stress-induced intestinal injury, which indicated that the FAE was more sensitive to stress-exposure. Mast cells as important effector cells in response to stress may participate in the process of epithelial hyper-permeability. Especially, we demonstrated that inhibition of mast cells and blocking the protease/PAR2 signaling obviously improved the barrier dysfunction in FAE.

Stress induced mild mucosal inflammation and visceral hypersensitivity in the rat model, with increased intraepithelial lymphocytes infiltration and reduced pain threshold to colorectal distention. It also has been similarly reported elsewhere^8, 13^. The negative effect of stress on intestinal barrier function may be one of the plausible mechanisms in triggering inflammation and pain^13, 14^. Indeed, as we shown, psychological stress lead to significant increase in both ileal and colonic epithelial permeability, such as decreased transepithelial resistance and elevated paracellular (FD4) and transcellular (FD40) macromolecular permeability. Meanwhile, levels of CRF, the key stress hormone, raised in serum and intestinal tissues under stress. CRF mainly expresses in the central nervous system, but also can be released locally by enterochromaffin cells, immune cells, regional sensory and sympathetic nerves in peripheral tissues^15–18^. Central and peripheral administration of exogenous CRF have been shown to mimic the stress-incuced intestinal dysfunctions including increasing ion and water secretion, mucus release and epithelial permeability^17^. Our *in vitro* observation also showed that CRF obviously disrupted the mucosal permeability in ileum and colon. It further verified that stress affect the intestinal barrier function involving the CRF pathway.

More importantly, we focused on the potentially different susceptibility of functionally distinct epithelium to stress-induced barrier disruption, including the ileal and colonic epithelium, and the VE and FAE. This idea was based on the fact that the structure and function of mucosal barrier differ in distinct segments and regions of gastrointestinal tract^12, 19^. On the whole, the ileal FAE showed a more significant elevation in paracellular and transcellular permeability compared with the ileal villous epithelium and colonic epithelium in rats exposed to stress *in vivo* and in mucosal tissues treated with CRF *in vitro*. However, there was no marked difference between ileal villous epithelium and colonic epithelium. It indicated that the FAE was more sensitive to stress.

It is reasonable that the FAE is more vulnerable to stress-induced barrier dysfunction, although it has not been addressed. Firstly, the FAE overlays the Peyer’s patches in the small intestine and typically contain microfold cell^10^; in addition, unlike the villous epithelium, it has less abundant goblet cells and different absorptive cells with absence of secretory component expression^10^. Therefore, the structure of FAE is entirely different from the VE. Secondly, the FAE is an important route for antigen sampling that differs from the normal VE^20^. It has been suggested to be associated with the unique biochemical properties of microfold cells, namely the highly efficient sampling of antigens^20, 21^. All these differences may form the basis of susceptibility of FAE to injury. More direct evidence showed that the first observable signs of Crohn’s disease are microscopic erosions originating from the FAE^1, 11, 22–24^. Although the FAE only account for a very small proportion of the whole intestinal epithelium^10^, it may be the key factor in regulating the mucosal and even systemic immune. These findings strengthened the hypothesis that the FAE might be the site of pathological changes onset in response to psychological stress. Consequently, more concerns should be paid to the FAE in permeability increasing and barrier dysfunction in conditions associated with stress including inflammatory and functional bowel diseases.

The mechanisms underlying permeability increasing has been widely studied in small intestinal and colonic epithelium under stress, including various neuroimmunological and neuroendocrine mechanisms^25, 26^. Particularly, MCs may play an important role in the prosess^7, 8, 26^. We verified that MCs degranulation, induced by compound 48/80, could disrupt the barrier of the FAE. However, very few researches addressed the possible relationship between MCs and stress induced FAE hyper-permeability. In our preliminary study, we demonstrated that there were increased tryptaes^+^-MCs and elevated expression of tryptase in parallel with its receptor PAR2 in FAE of the WAS rats, moreover, the tryptase and PAR2 level was positively related with the epithelial conductance of the FAE. Intraperitoneal injection with cromolyn, a MCs stabilizer, significantly improved the barrier function and upregulated the pain threshold. This further indicated that MCs activation involves in the process of stress-induced barrier disruption of the FAE, similarly to the VE^2, 7, 8^.

Mast cell act as a key effector in stress-induced intestinal epithelial barrier dysfunction^7, 27^. CRF is the important stress factor for the activation of MCs since both the 2 types of CRF receptors express on MCs^4, 28^. *In vivo* and *in vitro* administration of CRF resulted in MCs degranulating which could be prevented by blocking CRF receptors^2, 7^. Additionally, MCs, often found close to nerve fibers^29^, may also be directly activated by the nervous systems during stress and be independent with central and local CRF^6, 30^. It suggested that MCs could be a potential therapeutic target for barrier disruption of the FAE under stress. Our further *in vitro* investigation showed that MCs stabilization, protease inhibition and PAR2 blocking effectively eliminated the FAE hyper-permeability induced by CRF exposure. These findings are in line with previous evidences that PAR2 participated in the intestinal mucosal inflammation^31^. Tryptase may directly acted on the epithelial PAR2 and rearranged the tight junction proteins and perijunctional F-actin and eventually resulted in paracellular leakage and inflammation of the gut^18^. PAR2 activation may also promote the release of pro-inflammatory peptides such as calcitonin gene-related peptide and substance P, and lead to secretion of TNFα and IFNγ which in turn increase paracellular peameability and trigger intestinal inflammation^2, 18^. Then we tend to believe that elevated MCs and PAR2 signaling may also play a significant role in the FAE hyper-permeability under stress conditions.

In summary, stress-induced intestinal permeability increasing and barrier dysfunction was more sensitive and serious in the FAE than ileal villous epithelium and colonic epithelium. The FAE may be the initial site of lesion formation and depravation happened in the gut in conditions related to stress thus it need more attentions. MCs and the protease/PAR2 signaling played important roles in this process and promised to be potential therapeutic targets in preventing the stress-induced barrier disruption of the FAE.

## Methods

### Animals

Male Sprague-Dawley rats (aged 6 weeks, weighed 180–200 g; Experimental Animal Center, HUST, Wuhan, China) were used. Rats were housed under specific pathogen-free conditions with 12/12-hour light/dark cycle, temperature maintained at 23 °C and ad libitum access to standard rat chow and tap water. Before the following experiments, the rats were allowed to acclimatize to the new environment for one-week and get familiar to the experimenter. All experimental procedures were performed in accordance with the ethical guidelines of the International Association for the Study of Pain (IASP) and the Animal Management Rules of the Chinese Ministry of Health (Document No. 55, 2001), and approved by and the Animal Care and Use Committee, Union Hospital, Tongji Medical College, HUST, China (Approval ID 2013025).

### Water avoidance stress, sham stress and groups

Rats were randomly assigned to the sham stress group (SS, 12 rats), the water avoidance stress group (WAS, 12 rats) and the WAS + cromolyn sodium group (WAS + Crom, 12 rats). The rats in WAS + Crom group were injected with mast cell stabilizer cromolyn sodium (25 mg/kg) intraperitoneally 30 minutes before stress every day^32, 33^. As control, the SS group and WAS group were injected with equal amounts of physiological saline. The protocol of WAS and SS was described elsewhere previously^34^. That is, each rat was placed on a platform (8-cm length × 6-cm width) affixed at the center of a plastic cage (48-cm length × 48-cm width), which was filled with 25 °C warm water (about 1 cm below the platform), for 1 hour daily for 10 consecutive days. For the SS group, rats were placed on the same devices but without water in the cages. All the experiments were performed at 9–11 AM every day. The body weight was recorded each day before the WAS and SS, and the total number of stool pellets was counted during the 1-hour WAS or SS. After the 10-day stress, rats were anaesthetized by 2% pentobarbital sodium, then the serum and intestinal tissues including ileum VE, ileum FAE and colon were obtained and used for measurements. Visceral sensation was assessed before execution by abdominal withdrawal reflex (AWR) scores to colorectal distention described elsewhere by *Al-Chaer et al*.^35^. Inflammatory scores were graded according to the infiltration of intraepithelial lymphocyte and interstitial edema blindly by 2 investigators with a modified scale previously described by *Al-Chaer et al*. via H&E staining^35, 36^.

### Enzyme-linked immunosorbent assay for serum CRF

Serum samples were collected. CRF levels were detected through an enzyme linked immunosorbent assay (ELISA) kit (R&D Systems, USA) following the manufacturer’s instructions. Serum CRF was quantitated against a CRF standard curve.

### Real-time quantitative PCR analysis for intestinal CRF

The enterogenous CRF was assessed via mRNA expression in the intestine by means of real-time quantitative PCR (RT-qPCR). Total RNA in VE, FAE and colonic tissues were extracted using the Trizols Reagent (Invitrogen, Life Technologies). Complementary DNA was synthesized using the PrimeScript™ RT Master Mix Kit (TaKaRa) and then used for real-time quantitative PCR with a QuantiTect SYBR Green PCR Kit (QIAGEN) on a ROCHE LightCycler® 480 System. Primer sequences used for amplification were as follows: CRF forward 5′-GAAGAGAAAGGGGAAAGGCAAAGA-3′ and reserve 5′-GCGGTGAGGGGCGTGGAGTT-3′ (NM_031019); GAPDH forward 5′-ACCACAGTCCATGCCATCAC-3′ and reserve 5′-TCCACCACCCTGTTGCTGTA-3′ (NM_001289745). The relative expression of mRNA species was quantified by 2^−ΔΔCT^ method.

### Immunoflorescence staining

The intestinal tissues were fixed with 4% paraformaledehyde, embedded in paraffin and cut into slice. After routinely dewaxing and hydration, the slices were conducted antigen heat retrieval in citrate buffer (0.01 M, pH 6.0), and then blocked with 10% donkey serum (containing 0.3% Triton X-100) for 45 min at room temperature. Sections were incubated with the following primary antibodies overnight at 4 °C: mouse monoclonal anti-mast cell tryptase antibody (1:500, ab2378, Abcam) or rabbit monoclonal anti-PAR2 antibody (1:400, ab180953, Abcam). After washing with phosphate buffer saline (PBS) for 5 min * 3 times, sections were respectively stained with donkey anti-mouse or rabbit Alexa Fluor 488 ((IgG H&L) secondary antibodies (1:300, Invitrogen) for 60 min at room temperature, and then washed with PBS for 5 min * 3 times. Finally, DAPI (1 μg/ml, Beyotime Biotech, China) were used for nuclei staining. Images were viewed and captured using a confocal laser scanning microscope (Nikon, Japan) with excitation wavelength appropriate for Alexa Fluor (488 nm or 594 nm), and analyzed using NIS Elements Viewer Software (Nikon, Japan).

### Western blot analysis

The intestinal tissues were homogenized in a RIPA extraction buffer with a protease inhibitor cocktail (Roche) for 30 min. Proteins were quantified via a BCA protein assay kit (Beyotime Biotechnology, China), and then prepared in Laemmli buffer and boiled in water bath. Equal amounts of tissue lysates (50 mg) were loaded and separated on10% SDS-PAGE gels with constant voltage of 100 V for 100 min, and transferred to PVDF membranes with constant current of 300 mA for 80 min in ice bath. Membranes were subsequently blocked in 8% (w/v) non-fat dried milk for 1 h at room temperature, then incubated overnight at 4 °C with specific antibodies against tryptase (Abcam, ab109226, 1:2000), PAR2 (Abcam, ab154835, 1:1000), β-actin(Abbkine, A01010, 1:2000) and GAPDH (Abbkine, A01020, 1:2000). After fully washing, the membranes were probed with HRP-conjugated goat anti-rabbit or mouse antibody (1:5000, Pierce, USA) for 1 h at room temperature. Then, the protein bands were developed in the SuperSignal West Pico Substrate (Pierce, USA) and quantified by the FluorChem FC3 software (ProteinSimple, USA).

### Ussing chamber experiments and mucosal-to-serosal fluxes of macromolecules

The ileal VE, ileal FAE and colon from each group were stripped from the seromuscular layer in ice-bathed and oxygenated Krebs’ buffer. The mucosal tissues or patches were mounted on sliders with a circular hole (opening area 0.25 cm^2^) in the center, and covered this entire area of the hole which maintains the exposed area of 0.25 cm^2^. Then, the sliders were installed on U-type chambers for Ussing Chamber System, and the tissues were bathed in 37 °C oxygenated Krebs’ solution (5 ml on each side of the U-type chamber). The serosal buffer contained glucose as an energy source, while the mucosal buffer replaced by equivalent mannitol for osmotically balancing. The spontaneous potential difference (PD) was monitored via agar-salt bridges connected to calomel electrodes, and the appropriate short-circuit current (Isc) was added via Ag-AgCl electrodes to maintain a zero PD through an automatic voltage clamp (World Precision Instruments, USA). The TER and conductance was calculated from the spontaneous PD and Isc by means of Ohm’s law. After a 20-min equilibration period, the TER was recorded. Meanwhile, 1 mg/ml FD4 (or FD40), which represented the paracellular (or transcellular) macromolecular permeability, was added to the mucosal side of the U-type chambers, and sampled in the serosal side at 30-min intervals over a 3-h period. The FD4 (or FD40) intensity of each sample was measured by a Fluorescence Microplate Reader (BioTek Instruments, USA). The mucosal-to-serosal FD4 (or FD40) flux was determined by the increase of concentration of FD4 (or FD40) in the serosal side within 3 hours. Our preliminary results showed that tissues maintained alive for at least 4 hours with consistent transepithelial PD and Isc response to 10 mM forskolin adding to the mucosal chamber.

Furthermore, intestinal tissues from normal SD rats were used, mounted on Ussing Chambers and equilibrate for 20 min to reach a stabilized TER and Isc. Cromolyn (10^−4^ M), a mast cell stabilizer; aprotinin (10^−5^ M) or FUT-175 (10^−5^ M), the serine protease inhibitor were added to the serosal chamber 30 min prior to 0.25 μM CRF or 10 μg/ml compound 48/80 exposure. In order to clarify the role of PAR2 in the FAE barrier disruption, additional experiments with both specific PAR-2 agonists SLIGRL-NH2 (1 mM) and antagonist ENMD-1068 (5 mM) were further conducted. The TER, FD4 and FD40 flux were measured over a 3-h period as previously described.

### Chemicals and solutions

The Kreb’s solution contained (in mM, pH 7.2–7.4): 119 Na^+^, 4.7 K^+^, 1.2 H_2_PO_4_^−^, 25 HCO_3_^−^, 1.2 Mg^2+^, 2.5 Ca^2+^ and 11.1 Glucose, and all the reagents were from Sinopharm Chemical Reagent Co., Ltd, China. Human/rat corticotropin releasing factor (CRF) was acquired from R&D Systems Inc., MN, USA. Cromolyn sodium, compound 48/80, aprotinin, nafamostat mesylate (FUT-175), ENMD-1068, SLIGRL-NH2, FD4 and FD40 were obtained from Sigma-Aldrich Co., LLC, USA.

### Data expression and statistical analysis

The data were expressed as the mean ± standard deviation. The normality of the data, as well as the homogeneity of variance, was tested. Accordingly, the parametric one-way analysis of variance (ANOVA) or non-parametric Kruskal-Wallis tests was used for data analyzing, and the least significant difference (LSD) test or Dunnett’s T3 test was used for multiple comparisons. The correlation between tryptase/PAR2 expression and epithelial conductance was analyzed by means of Pearson correlation analysis. *P*-values less than 0.05 were considered statistically significant.

## Electronic supplementary material

Supplementary Information
