# Supplementary material for: Stress induces more serious barrier dysfunction in follicle-associated epithelium than villus epithelium involving mast cells and protease-activated receptor-2
Source: Sci Rep. 2017 Jul 10;7:4950. doi: 10.1038/s41598-017-05064-y (PMC5503989; doi:10.1038/s41598-017-05064-y)

Stress induces more serious barrier dysfunction in follicle-associated epithelium than villus epithelium involving mast cells and protease-activated receptor-2

Authors:

Lei Zhang[1], Jun Song[1], Tao Bai[1], Wei Qian[1] & Xiao-Hua Hou[1*]

Affiliations:

[1] Division of Gastroenterology, Union Hospital, Tongji Medical College, Huazhong University of Science and Technology, Wuhan, China

Correspondence:

Xiao-Hua Hou, Ph.D., M.D.

Division of Gastroenterology, Union Hospital, Tongji Medical College, Huazhong University of Science and Technology, 1277 Jiefang Road, Wuhan, 430022, China

Tel: +86-027-85726057; Fax: +86-027-85726057

E-mail: houxh@medmail.com.cn

Supplementary Figure S1

The full scan images of the immunoblots. In order to minimize the amount of antibodies, the PVDF membranes for immunoblots were cut into strips which are necessary for analysis. Selected data were shown in the manuscript and scans of entire PVDF strips are provided.

Figure 3J

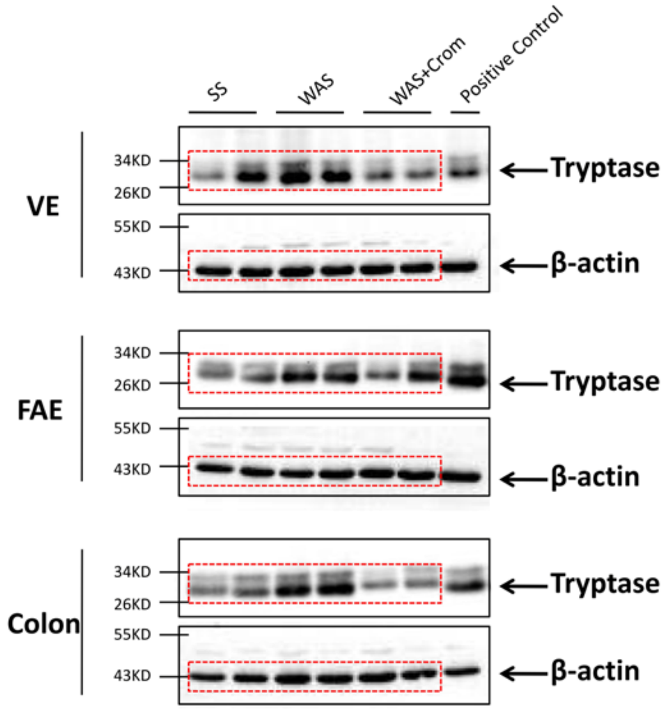

Figure 4G

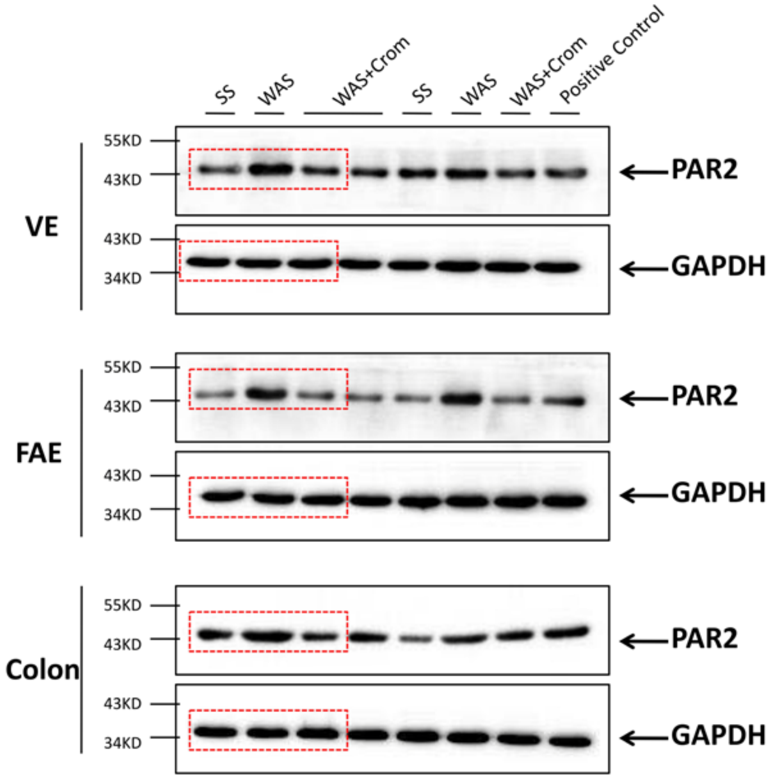

Supplement: Supplementary file 1 — Supplementary Information [file 41598_2017_5064_MOESM1_ESM.pdf]
